# Gossypol Promotes Bone Formation in Ovariectomy-Induced Osteoporosis through Regulating Cell Apoptosis

**DOI:** 10.1155/2018/3635485

**Published:** 2018-12-13

**Authors:** Jinqian Liang, Chong Chen, Hongzhe Liu, Xiangyang Liu, Zheng Li, Jianhua Hu, Hong Zhao

**Affiliations:** ^1^Department of Orthorpaedic Surgery, Peking Union Medical College Hospital, Beijing 100730, China; ^2^Department of Orthopedics, People's Hospital of Hunan Province, Hunan 410005, China

## Abstract

Osteoporosis is among the most common forms of age-related diseases, especially for females, which has been a grave public health problem. Drug therapies have shown promising outcomes to promote bone formation and bone density. This study identified a novel potential drug, gossypol, for the treatment of osteoporosis. Treatments of ovariectomy-induced osteoporosis mice with gossypol significantly increased serum osteocalcin and osteoprotegerin (OPG) levels; meanwhile they decreased serum RANKL levels. Microcomputed tomography (microCT) analysis showed that treatment of gossypol improved bone density and strength and decreased bone postyield displacement for both medullar and cortical bones.* In vitro* experiments also showed that gossypol increased cell viability in a time- and dose-dependent manner. Furthermore, incubation of the osteoblast MC3T3-E1 cells with gossypol inhibited cell apoptosis through intrinsic apoptotic pathway as evidenced by the Annexin V/PI assay, TUNEL assay, biochemical analysis, and western blot assays. Moreover, the classical Wnt/*β*-catenin signaling pathway was found to be regulated by gossypol treatments. Inhibition of Wnt/*β*-catenin signaling reversed the prevention effects of gossypol in osteoporosis. Our findings provided novel clues for the treatment of osteoporosis in clinic.

## 1. Introduction

Osteoporosis is one of the most common causes for a broken bone among the elderly [1], which may result from lower bone mass and greater bone loss [2]. Osteoporosis may occur due to aging and a variety of diseases or treatments including alcoholism, hyperthyroidism, kidney failure, and surgical removal of the ovaries [3, 4]. The underlying mechanism of all kinds of osteoporosis is an imbalance between bone resorption and bone formation, resulting from mesenchymal stem cells (MSC) biasing from osteoblast and towards the marrow adipocyte lineage [5, 6]. Of note, estrogen strongly determines the rate of bone resorption, lack of which increases bone resorption and decreases the deposition of new bones that take place in weight-bearing bones [7]. In addition to hormones, calcium metabolism appears to be one of the most important factors in bone turnover. Deficiency of calcium and vitamin D may lead to impaired bone deposition [8, 9].

Lifestyle prevention and nutrition supplement with calcium and vitamin D are two main solutions to relieve osteoporosis [10]. Besides, medication intervene is another effective way, such as bisphosphonates, which decreases the risk of future fractures in individuals who have already sustained a fracture after osteoporosis [11]. However, for those with osteoporosis but not with a fracture evidence, risedronate or etidronate is not supportive for a reduction in fracture risk [12]. Fluoride supplement is not effective enough for postmenopausal osteoporosis since it increases bone density but does not decrease the risk of fractures [13]. Thus, there is still a long way to go to cure osteoporosis.

Gossypol is a natural product derived from the genus* Gossypium*, which is a phenolic aldehyde and acts as an inhibitor of various dehydrogenase enzymes [14]. Besides its putative contraceptive function, gossypol is a well-known antimalarial and a potential anticancer drug [15]. Recently, Saleh et al. reported the critical role of gossypol in cell inflammation and oxidative stress [16]. Chen et al. demonstrated gossypol could stimulate the opening of a Ca^2+^- and Na^+^- permeable but Ni^2+^- and Co^2+^- impermeable pore in endothelial cells [17], hinting that gossypol may be related to the progression of osteoporosis. However, whether it is effective for bone formation during the development of osteoporosis has never been discovered. Herein, we studied the role of gossypol in osteoporosis induced by ovariectomy* in vivo* and* in vitro*. Histology, microCT and blood index were included and mouse MC3T3-E1 cell line was used for functional analysis.

## 2. Materials and Methods

### 2.1. Animals

This study was approved by the Ethic Committee of Department of Orthopedic, People's Hospital of Hunan Province. A total of 20 C57BL/6 mice (age: 8 weeks, male) were purchased from Model Animal Research Center of Nanjing University. All of the mice were housed under standard conditions of 12h: 12h dark/light cycles. Gossypol was purchased from APExBIO Co. (Catalog number: N2135, NY, USA) and dissolved in DMSO with a concentration of 15mg/ml. All mice underwent oophorectomy and then were randomly divided into two groups: control group (*i.p.* injection with DMSO) and gossypol (*i.p.* injection with gossypol with a dose of 15mg/kg). The injection was continued every day for four weeks. Afterwards, mice were sacrificed after anesthesia with 7% pentobarbital sodium and the thighbone of each mouse was harvested.

### 2.2. Cell Culture

MC3T3-E1 cell line deprived from neonate C57BL/6 mice was purchased from American Type Culture Collection (CRL-2593, Massachusetts, USA) and cultured in DMEM (Gibco, NY, USA) supplemented with 10% fetal bovine serum (FBS, Gibco). Wnt/*β*-catenin signaling pathway inhibitor, ICG-001, was purchased from Selleck (Shanghai, China) and dissolved in DMSO with a concentration of 50mM. Cells were treated with gossypol with a concentration of 20nm for 24h, unless otherwise stated.

### 2.3. Histology

The thighbone from each mouse was fixed in 10% paraformaldehyde and decalcified with a Sigma-Aldrich decalcifying reagent. After dehydrating for 18 hours, the tissues were embedded with paraffin and cut into 5*μ*m slices for hematoxylin and eosin (H&E) staining. Five random fields were selected and photographed under a Nikon (Japan) light microscope to calculate bone formation and osteoporosis.

### 2.4. Elisa Assays

Blood samples from each mouse were collected for biochemical analyses. The serum osteocalcin levels were assessed with a Life Technology Enzyme-linked immunosorbent assay kit (Elisa, CA,USA) according to the manufactures' instructions. Serum RANKL and osteoprotegerin (OPG) levels were determined with Elisa kits from R&D system (Minneapolis, MN, USA) as per the protocols.

### 2.5. MicroCT Analysis

A microcomputed tomography (microCT) system (Bassersdorf, Switzerland) and the corresponding analysis software were applied for the microCT analysis. Briefly, the samples were harvested and X-rays previewing was performed using a 1024× 256 element CCD array, which was fixed with a Compaq/hp *α*-Station operating in an open VMS environment. Samples were scanned on femurs with medium resolutions. After each scanning, the system got an image dataset by 1024 two-dimensional axial slices at the midshaft region of femurs. It took about 5 minutes for each bone sample.

### 2.6. Induction of MC3T3-E1 Cells Differentiation into Osteoblasts

To differentiate into osteoblasts, MC3T3-E1 cells were seeded on 24-well plates at a density of 6.5×10^4^ cells and cultured in *α*-MEM supplemented with 10 mM *β*-glycerol phosphate, 50 *μ*g/ml ascorbic acid, and 10 *μ*M dexamethasone for 21 days. The media of cultured cells were replaced every 3 days.

### 2.7. Cell Viability Assays

Cell viability was assessed with the methylthiazoletetrazolium (MTT) assay. Briefly, cells were treated with gossypol for different dose (0, 1, 5, 10, and 20nM) and various time periods (0, 6, 12, 18, and 24h). Afterwards, cells were trypsinized and reseeded in triplicate in a 96-well plate at an initial density of 4,000 cells/well. Then, cells were added with 10*μ*l of MTT solution (5 mg/mL) per well and incubated for 2h at 37°C; the absorbance of each well was recorded at 570 nm. Cell viability was calculated as the cell number ratio of experimental groups to control cells.

### 2.8. Apoptotic Analysis

The Annexin V/ PI assay was conducted as per the manufacturer's instructions (Invitrogen, NY, USA). Briefly, MC3T3-E1-differentiated osteoblast cells were treated with gossypol with a concentration of 20nM for 24 hours. Afterwards, cells were washed with prechilled PBS, trypsinized with trypsin for 1 min, and resuspended in 100*μ*l of binding buffer supplemented with 2.5*μ*l FITC conjugated Annexin V and 1*μ*l PI (100 *μ*g/ml). Then, cells were shaken at room temperature for 15 min in a void of light. A total of 10, 000 cells were collected and assessed by flow cytometry (BD Biosciences).

### 2.9. Terminal Deoxynucleotidyl Transferase dUTP Nick End Labeling (TUNEL) Assays

The TUNEL assays were performed according to the protocols (Vazyme, Nanjing, China). Cells were stimulated with gossypol for 24 hours and then washed with ice-cold PBS. After incubation with lysis buffer for 10 min on ice, the cell pellet was collected with low-speed centrifugation (1000g, 5 min, 4°C). Slides with adherent cells were covered with 50 ul terminal deoxynucleotidyl transferase reaction mixture for 60 min at 37°C in darkness. After being stopped with SSC for 15 min, nuclei were stained and visualized with DAPI staining. Five random fields were photographed with the Nikon light microscope.

### 2.10. Relative Caspase Activities

The activities of caspase-3, caspase-8, and caspase-9 were assessed with caspase activity kits (Beyotime, Nantong, China). Briefly, cells were treated with gossypol for 24 hours and then collected by low-speed centrifugation (1000g, 5 min, 4°C). A total of 100*μ*g proteins from each sample were added to a 96-well plate and mixed with an aliquot of 80*μ*l reaction buffer as well as caspase-3, caspase-8, and caspase-9 substrates (2 mM). After incubation at 37°C for 4h, caspase activities were determined by the microplate reader at an absorbance of 450 nm and the data were normalized to the control cells.

### 2.11. Western Blot Analysis

Osteoblast cells were treated with gossypol with a concentration of 20nM for 24 hours. Afterwards, cells were harvested with lysis buffer (NP-40, Beyotime, Nantong, China) and a total of 10*μ*g proteins were loaded up to a 10% SDS-PAGE gel. After being transferred to a nitrocellulose membrane, the proteins were blocked with 5% skim milk in TBST at room temperature for 1 hour and incubated with primary antibodies at 4°C overnight. The primary antibodies against osteocalcin (ab13420), osterix (ab209484), Runx2 (ab23981), Wnt1 (ab15251), *β*-Catenin (ab16051), and COL1A1 (ab90395) were purchased from Abcam (Cambridge, USA) and diluted into 1:1000 for use. The primary antibodies against ODF (sc-390152), AKP (sc-271431), GAPDH (sc-47724), and secondary antibodies (1:5000) were commercially from Santa Cruz Biotechnology (Santa Cruz, CA, USA). The primary antibody against TGF*β*1 was from Proteintech Inc. (21898-1-AP, 1:1000) (Wuhan, China).

### 2.12. Real-Time Polymerase Chain Reaction (RT-PCR)

Total RNAs from cells were extracted with a standard Trizol solution (Thermo Scientific, NY, USA). RNAs were quantified by Nanodrop 2000 by collecting the OD260 and OD280 absorbance and an amount of 1000ng RNA was reversely transcribed into cDNA with Prime Script TM Master Mix (Takara, Japan). qRT-PCR was performed with SYBR Premix EX Taq TM II (Takara) on the real-time PCR detection system ABI7500 (Thermo Fisher Scientific, CA, USA). GAPDH was included as an inner control and gene mRNA expression was calculated by 2^−ΔΔCt^ method. The qPCR protocol was shown as follows: initial denaturation at 95°C for 5 min, followed by 45 repeats of a three-step cycling program consisting of 10 sec at 95°C (denaturation), 10s at 60°C (primer annealing), and 10 sec at 72°C (elongation) and a final extension step for 10 min at 72°C. The primers used were listed in Table 1.

### 2.13. Statistical Analysis

GraphPad Prism (GraphPad Software, La Jolla, CA, USA) software was included for statistical analysis. Data were shown as mean ± standard deviation (SD). The two-tailed Student's* t*-test was used to compare means between two groups and one-way analysis of variance (ANOVA) was used for comparisons among multiple groups (≥3 groups), followed by a LSD post hoc test. Chi-square or Fisher's exact test was used to compare proportion differences of categorical variables. Differences were considered statistically significant when a two-sided* p *value was less than 0.05.

## 3. Results

### 3.1. Gossypol Promotes Bone Formation in Ovariectomy-Induced Osteoporosis* In Vivo*

**Figure 1:**
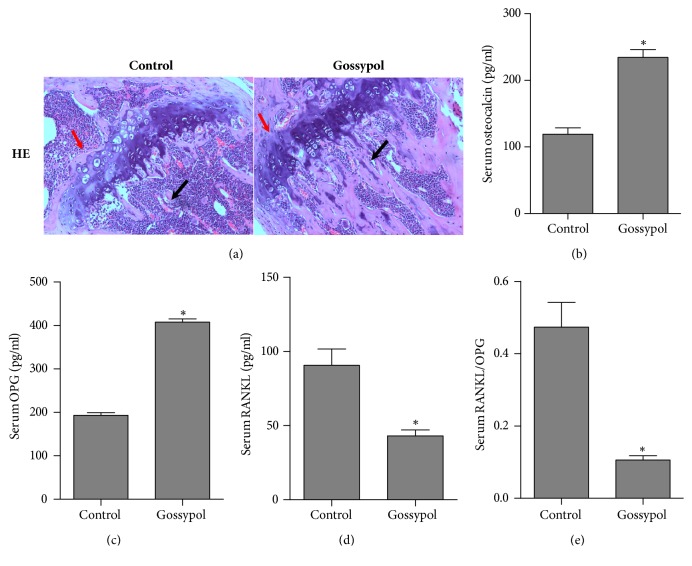
**Gossypol promotes bone formation in ovariectomy-induced osteoporosis* in vivo.*** (a) Mice underwent ovariectomy in the presence or absence of gossypol injection and the thighbone of each mouse was collected for H&E staining. Red arrow means trabeculae of epiphyseal and black arrow means trabeculae in metaphyseal regions. (b) Serum osteocalcin level of each mouse in control and gossypol injection groups was determined with Elisa methods. (c) Serum OPG levels were assessed with Elisa methods for the mice injected with solvent or gossypol. (d) Serum RANKL levels of mice from two groups were measured with Elisa methods. (e) Serum RANKL/OPG levels were assessed by the ratio of serum RANKL and serum OPG levels. Each experiment was repeated for three times. Data were presented as mean ±SEM. *∗P<*0.05 versus control.

To evaluate the effects of gossypol on bone formation, we first performed histological analysis with ovariectomy-induced osteoporosis mice. As shown in Figure 1(a), gossypol-injected mice showed relieved osteoporosis as characterized by a remarkable increase in the trabeculae of epiphyseal (red arrows) and metaphyseal regions (black arrows) when compared with the control mice. The increased thickness of cortical bone was also observed in the metaphyseal region in the gossypol-treated group (data not shown). Furthermore, we also analyzed several serum chemicals with Elisa methods in both groups. It was shown that the serum osteocalcin levels in gossypol-injected mice were significantly higher than the control counterpart (Figure 1(b)). Furthermore, the serum OPG levels in the experimental mice were increased to 2-fold of the control group (Figure 1(c)) and the serum RANKL levels were decreased by approximate 50% (Figure 1(d)), causing the ratio of RANKL to OPG to drop to 25% in the gossypol-injected mice in comparison with the control group (Figure 1(e)).

**Figure 2:**
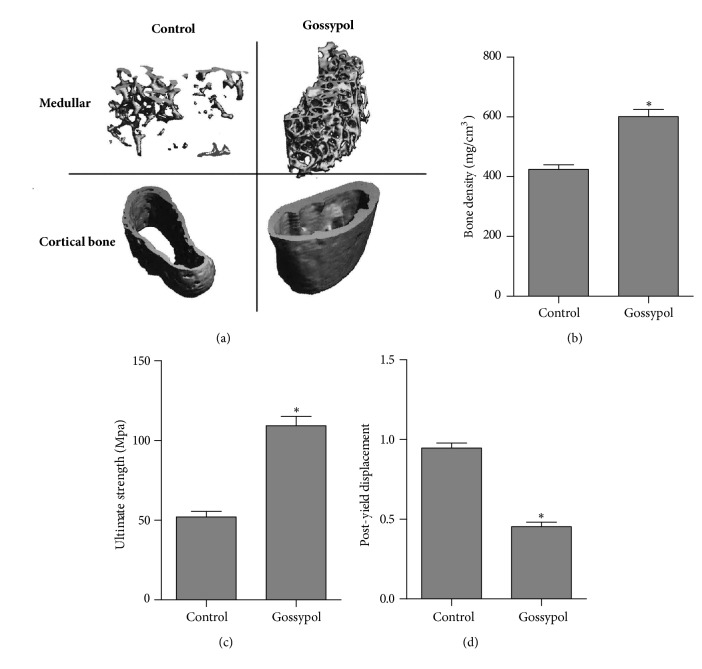
**Gossypol increased bone strength after ovariectomy. **(a) MicroCT was used to scan the thighbone of each mouse in control and gossypol group. (b) Bone density of each mouse was assessed in control and gossypol group. (c) Ultimate strength of each mouse was determined in control and gossypol group. (d) Postyield displacement of each mouse was measured in control and gossypol group. Each experiment was repeated for three times. Data were presented as mean ±SEM. *∗P<*0.05 versus control.

Next, microCT scanning was performed and found that, as compared with vehicle-treated osteoporosis mice, gossypol-injected mice had higher medullar and cortical bone density (Figure 2(a)). Furthermore, the bone density of control mice was approximately 400mg/cm^3^, which was significantly lower than that in the gossypol-treated mice (600mg/cm^3^) (Figure 2(b)). It was further shown that gossypol treatment rescued ovariectomy-induced bone strength loss by almost 50% as compared to the control group (Figure 2(c)). Moreover, the postyield displacement of gossypol-injected mice was also dropped to 50% of the control counterparts (Figure 2(d)). These data together with Figure 1 suggested that gossypol promotes bone formation in ovariectomy-induced osteoporosis mice.

### 3.2. Gossypol Promotes Cell Viability in Osteoblast Cell Line MC3T3-E1 In Vitro

**Figure 3:**
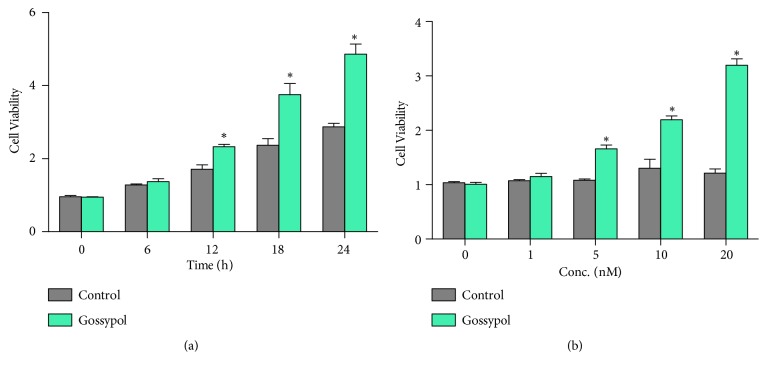
**Gossypol promotes cell viability in osteoblast cell line MC3T3-E1* in vitro.*** (a) Cell viability was assessed in cells treated with DMSO or gossypol at different time intervals. (b) Cell viability was assessed in cells treated with DMSO or gossypol with various concentrations. Each experiment was repeated for three times. Data were presented as mean ±SEM. *∗P<*0.05 versus control.

To identify the specific role of gossypol in bone formation, we further examined the cell viabilities of osteoblast cell line MC3T3-E1 upon gossypol treatment. After differentiation, MC3T3-E1 cells were treated with gossypol for various time periods. It was shown that the cell viability was increased at the time checkpoint 12h and the further upregulated with the prolonged time periods 18h and 24h (Figure 3(a)). Moreover, higher doses of gossypol increased cell viability in a dose-dependent manner as compared with control cells (Figure 3(b)). These results showed gossypol promoted cell viability in osteoblasts in a time- and dose-dependent manner.

### 3.3. Gossypol Inhibits Cell Apoptosis in Osteoblast Cells In Vitro

**Figure 4:**
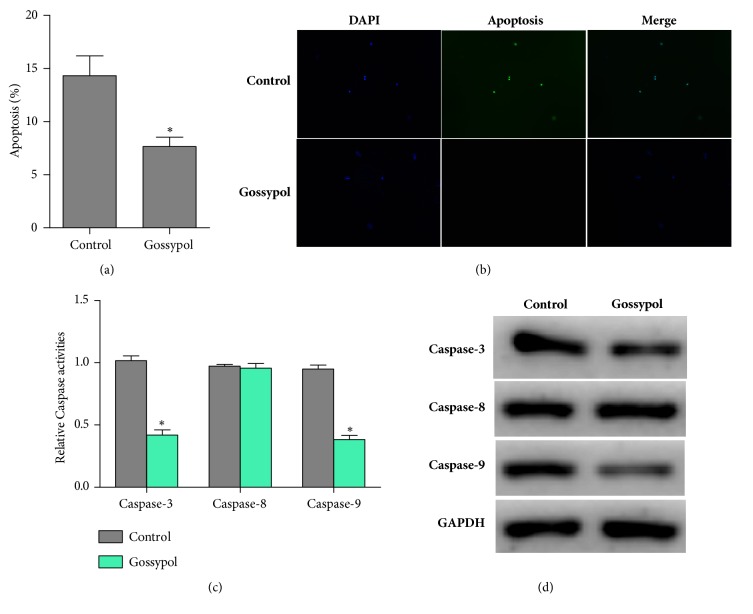
**Gossypol inhibits cell apoptosis in osteoblast cells* in vitro.*** (a) Cell apoptosis was determined in MC3T3-E1 cells treated with gossypol for 48h with a concentration of 20nm. (b) TUNEL assay was performed to assess the cell apoptosis in cells treated with gossypol. Five random fields were selected for calculation. (c) Relative activities of caspase-3, caspase-8, and caspase-9 in cells treated with gossypol. (d) Western blot assays were performed to examine the expression of caspase-3, caspase-8, and caspase-9 in MC3T3-E1 cells in response to gossypol treatments. Each experiment was repeated for three times. Data were presented as mean ±SEM. *∗P<*0.05 versus control.

Next, we examined the effects of gossypol on cell apoptosis. To this end, MTT assays were performed, which showed that the apoptotic rate of MC3T3-E1 cells in gossypol-treated group was notably decreased by 8% compared with the control cells (Figure 4(a)). TUNEL assay also revealed that the apoptotic cells were rescued by gossypol stimulation* in vitro* (Figure 4(b)). Moreover, we primitively checked the two signaling pathways of apoptosis: intrinsic and extrinsic pathways with RT-PCR and western blot assays. It was shown in Figure 4(c) that the relative activities of caspase-3 and caspase-9, which were both involved in the intrinsic pathway, were remarkably decreased by more than 50% in gossypol-treated cells, while that of caspase-8, a critical caspase involved in extrinsic pathway, remained stable. Similarly, gossypol treatment led to the protein levels of caspase-3 and caspase-9 dropping significantly, while causing no effects to that of caspase-8. All of these data suggested gossypol inhibited cell apoptosis in MC3T3-E1 cells through intrinsic apoptotic pathway.

### 3.4. Gossypol Promotes Bone Formation through Wnt/*β*-Catenin Signaling Pathway

**Figure 5:**
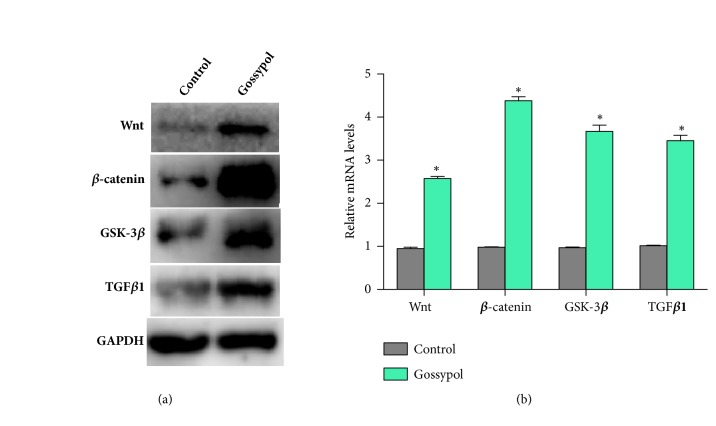
**Gossypol treatments in MC3T3-E1 cells increases Wnt/** **β** **-catenin signaling pathway.** (a) Western blot assays examined the protein levels of Wnt, *β*-catenin, GSK-3*β*, and TGF*β*1 in cells treated with gossypol. (b) qRT-PCR assays examined the mRNA levels of Wnt, *β*-catenin, GSK-3*β*, and TGF*β*1 in cells treated with gossypol. Each experiment was repeated for three times. Data were presented as mean ±SEM. *∗P<*0.05 versus control.

To elucidate the detailed mechanism of how gossypol regulates cell apoptosis, several signaling pathways were examined, among which Wnt/*β*-catenin showed the best response to gossypol treatments. Western blot analysis showed that gossypol treatment significantly upregulated the protein levels of Wnt, *β*-catenin, GSK-3*β*, and TGF*β*1 (Figure 5(a)). Furthermore, the mRNA levels of Wnt, *β*-catenin, GSK-3*β*, and TGF*β*1 in gossypol-treated group were also increased by more than 2-fold compared to those in the control cells (Figure 5(b)). These data showed gossypol promoted Wnt/*β*-catenin signaling pathway in MC3T3-E1 osteoblast cells.

**Figure 6:**
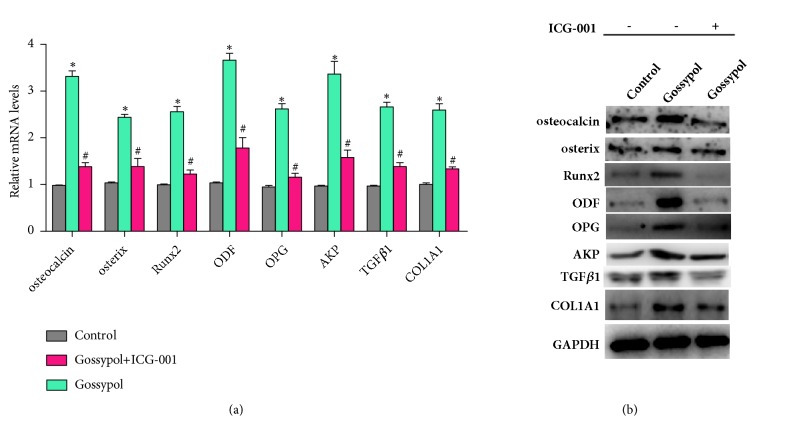
**Inhibition of Wnt//** **β** **-catenin signaling pathway reversed the effects caused by gossypol* in vitro.*** (a) mRNA levels of bone formation related genes in MC3T3-E1 cells treated with gossypol in presence or absence of ICG-001. *∗P<*0.05, gossypol versus control. ^#^*P<*0.05, gossypol+ ICG-001 versus gossypol. (b) Protein levels of bone formation related genes (osteocalcin, osterix, Runx2, ODF, OPG, AKP, TGF*β*1, and COL1A1) in MC3T3-E1 cells treated with gossypol in presence or absence of ICG-001. Each experiment was repeated for three times. Data were presented as mean ±SEM.

Next, we intervened the Wnt/*β*-catenin signaling pathway with its inhibitor ICG-001 and examined the expression of related genes. As shown in Figure 6(a), gossypol treatment increased the mRNA levels of bone formation related genes, such as osteocalcin, osterix, Runx2, ODF, OPG, AKP, TGF*β*1, and COL1A1 in comparison with the control cells. Likewise, all of these proteins were upregulated by gossypol treatment and reversed after coincubation with Wnt/*β*-catenin pathway inhibitor ICG-001 (Figure 6(b)). All of these observations demonstrated that gossypol promoted bone formation through activating Wnt/*β*-catenin pathway.

## 4. Discussion

Similar to the senile osteoporosis that is also caused due to the lack of estrogen, ovariectomy could lead to the imbalance of bone turnover, causing higher bone resorption and lower bone formation, and eventually induces osteoporosis [2]. Promising drug therapies for ovariectomy-induced osteoporosis or senile osteoporosis have improved the living situations for the patients; however, certain drugs are not suitable for all patients and may cause some side effects for some individuals [18]. Thus, it is still necessary to discover novel drugs for the treatment of osteoporosis. Here, we showed that a new natural compound, gossypol, promoted bone formation in osteoporosis mice by inhibiting cell apoptosis via activating Wnt/*β*-catenin pathway.

With the aid of microCT and histology methodology, it was initially found that gossypol significantly increased both cortical and medullar bone strength. The treatments of osteoporotic mice with gossypol also showed convincing turnover of osteoporosis as evidenced by the serum biochemical analysis. All these* in vivo* and* in vitro *data confirmed the beneficial roles of gossypol against osteoporosis. The activation of osteoblasts is regulated by multiple molecular signals, of which RANKL (receptor activator of nuclear factor kappa-B ligand) is the most important one. RANKL is produced by osteoblasts and lymphocytes and stimulates RANK. Osteoprotegerin (OPG) competes with RANK to bind to RNAKL, suppressing its ability to improve bone resorption [19]. Due to their important functions in osteoporosis, serum levels of osteocalcin, OPG, and RANKL were determined with Elisa kits and the results showed that gossypol treatment could promote bone formation through increasing their serum secretions. Therefore, it could be concluded that gossypol protected against osteoporosis in mice.

Cell apoptosis represents a critical biological process for disease development [20]. In this study, it was found that gossypol treatments decreased osteoblast cell viability. The inhibition of cell apoptosis by gossypol was mainly executed through activation of caspase-3 and caspase-9, two critical executors of intrinsic apoptotic caspases [21, 22]. Hence, gossypol promoted bone formation mainly through inhibiting osteoblast cell apoptosis.

Previous studies have demonstrated that Wnt/*β*-catenin signaling is crucial for bone formation in human osteoporosis [23]. Activation of the Wnt/*β*-catenin could increase trabecular and cortical bone mass through activation of mesenchymal stem cells, blockade of osteoblast apoptosis, and induction of osteoblast differentiation [24]. Furthermore, this pathway is an enticing target for drug development of osteoporosis and even other skeletal health problems [25]. Similar to those previous studies, we found that gossypol treatment in MC3T3-E1 cells increased Wnt/*β*-catenin signaling and inhibition of this pathway with its specific inhibitor and ICG-001 reversed the protective effects of gossypol on bone formation. The regulation of osteoblast cell apoptosis by Wnt/*β*-catenin signaling, therefore, might pave the way for gossypol-mediated bone formation.

## 5. Conclusion

Our study identified gossypol as a potential therapeutic drug for osteoporosis. Gossypol promoted bone formation by activating the Wnt/*β*-catenin signaling and thereby decreasing cell apoptosis. These results provided novel insights into the treatment of osteoporosis in clinic.

## Figures and Tables

**Table 1:** Primers used in this study.

Gene	Forward	Reverse
Osteocalcin	ACACTCCTCGCCCTATTG	GATGTGGTCAGCCAACTC
Osterix	ACACTCCTCGCCCTATTG	TAGAAGGAGCAAGGGGACAGAA
Runx2	TCTGGAAAAAAAAGGAGGGACTATG	GGTGCTCGGATCCCAAAAGAA
ODF	CACCTGGTTGCTGACTAATTGAGA	CTTGCTGTCCGACCAAATA
OPG	AACGGCAACACAGCTCACAAGAAC	TGCTCGAAGGTGAGGTTAGCATGT
Wnt	CGATGGTGGGGTATTGTGAAC	CCGGATTTTGGCGTATCAGAC
AKP	CCAACTCTTTTGTGCCAGAGA	GGCTACATTGGTGTTGAGCTTTT
GSK-3*β*	ATGGCAGCAAGGTAACCACAG	TCTCGGTTCTTAAATCGCTTGTC
TGF*β*1	CCACCTGCAAGACCATCGAC	CTGGCGAGCCTTAGTTTGGAC
COL1A1	CACCAATCACCTGCGGTACAGAA	CAGATCACGTCATCGCACAAC
*β*-catenin	AAAGCGGCTGTTAGTCACTGG	CGAGTCATTGCATACTGTCCAT
GAPDH	GTGGACATCCGCAAAGAC	AAAGGGTGTAACGCAACTA

## Data Availability

The data used to support the findings of this study are available from the corresponding author upon request.
